# Suppressive Effects on the Immune Response and Protective Immunity to a JEV DNA Vaccine by Co-administration of a GM-CSF-Expressing Plasmid in Mice

**DOI:** 10.1371/journal.pone.0034602

**Published:** 2012-04-06

**Authors:** Hui Chen, Na Gao, Dongying Fan, Jiangman Wu, Junping Zhu, Jieqiong Li, Juan Wang, Yanlei Chen, Jing An

**Affiliations:** Department of Microbiology, School of Basic Medical Sciences, Capital Medical University, Beijing, People's Republic of China; Albany Medical College, United States of America

## Abstract

As a potential cytokine adjuvant of DNA vaccines, granulocyte-macrophage colony–stimulating factor (GM-CSF) has received considerable attention due to its essential role in the recruitment of antigen-presenting cells, differentiation and maturation of dendritic cells. However, in our recent study of a Japanese encephalitis virus (JEV) DNA vaccine, co-inoculation of a GM-CSF plasmid dramatically suppressed the specific IgG response and resulted in decreased protection against JEV challenge. It is known that GM-CSF has been used in clinic to treat neutropenia for repopulating myeloid cells, and as an adjuvant in vaccine studies; it has shown various effects on the immune response. Therefore, in this study, we characterized the suppressive effects on the immune response to a JEV DNA vaccine by the co-administration of the GM-CSF-expressing plasmid and clarified the underlying mechanisms of the suppression in mice. Our results demonstrated that co-immunization with GM-CSF caused a substantial dampening of the vaccine-induced antibody responses. The suppressive effect was dose- and timing-dependent and likely related to the immunogenicity of the antigen. The suppression was associated with the induction of immature dendritic cells and the expansion of regulatory T cells but not myeloid-derived suppressor cells. Collectively, our findings not only provide valuable information for the application of GM-CSF in clinic and using as a vaccine adjuvant but also offer further insight into the understanding of the complex roles of GM-CSF.

## Introduction

In recent years, DNA vaccines have attracted much attention for their ability to induce both humoral and cellular immune responses. Nevertheless, despite their significant advantages, DNA vaccines have only shown limited success in animal models because of their low immunogenicity. Thus, to improve the efficacy of DNA vaccines, a number of strategies, especially the use of cytokine adjuvants, have been actively explored. Moreover, co-immunization strategies with plasmids expressing cytokines, such as interleukin (IL)-2, IL-4, IL-12, interferon (IFN)-γ, tumor necrosis factor (TNF)-α and granulocyte-macrophage colony–stimulating factor (GM-CSF) [1], [2], [3], [4], [5], or plasmids expressing co-stimulatory molecules [6] have been evaluated extensively with numerous DNA vaccines. Among these cytokines, GM-CSF has been the primary choice for many studies due to its essential role in the recruitment of antigen-presenting cells (APCs) and in the differentiation and maturation of dendritic cells (DCs) [5], [7], [8], [9]. However, as an adjuvant, various roles of GM-CSF have been reported: it appeared to help generate an immune response in some studies but had no effect or even an inhibitory effect in others. For example, in our recent study on a Japanese encephalitis virus (JEV) DNA vaccine, we unexpectedly found that co-injection of the GM-CSF plasmid significantly suppressed the specific IgG response and led to decreased protection against JEV challenge [10]. Similarly, a suppressive effect of the GM-CSF plasmid was also observed by a study of a human immunodeficiency virus (HIV) DNA vaccine, in which high levels of type I IFN at the local inoculation site involved in this process were discovered [11]. In a multi-center randomized trial of a melanoma vaccine, the CD8^+^ and CD4^+^ T cell responses were lower with the co-administration of recombinant GM-CSF [12]. Remarkably, a randomized study of 133 cancer patients treated with a trivalent influenza vaccine with GM-CSF administered at a dose of 250 µg also failed to show an increased immune response [13]. These data indicate that co-administration of GM-CSF failed to amplify the immune response and it even had a suppressive effect, which challenges the potential of using GM-CSF as a vaccine adjuvant and raises concerns that it might be harmful.

It is known that the GM-CSF receptor is expressed on CD34^+^ progenitor cells, all myeloid lineages and vascular endothelial cells. GM-CSF can promote myeloid differentiation, and it was initially discovered as a factor with the ability to generate both granulocytes and macrophage colonies from bone marrow precursor cells. Until now, GM-CSF has been routinely used in clinic to treat neutropenia for repopulating myeloid cells in post-chemo/radiotherapy cancer patients or post-bone marrow transplantation patients [14]. However, GM-CSF showed opposite functions as an adjuvant or therapeutic agent. Based upon the contradictory findings regarding immune response and clinical outcome, the use of GM-CSF in select treatments and adjuvant candidates must be performed with a great deal of caution. Thus, to provide more useful information for safe and reasonable clinical application, it is necessary to investigate the properties of the suppressed effects and to clarify the mechanism behind the phenomenon.

Recent studies have demonstrated that several factors contribute to immune suppression, including DCs, regulatory T cells (Tregs) and myeloid-derived suppressor cells (MDSCs). DCs are professional APCs that process and present foreign- as well as self-antigens and secrete a variety of cytokines and chemokines to initiate and regulate immune responses to ensure immunological homeostasis [15], [16]. The strength and nature of the immune response elicited by DCs depend on certain factors, including the type of antigens and the subset and maturation status of DCs. Generally, upon antigen presentation, mature DCs potently induce an efficient primary T cell response and differentiation of effector cells, whereas immature or semimature DCs are related to immune tolerance through the induction of Tregs or deletion of responding T cells [17], [18]. The maturation status of DCs is determined by the expression of surface molecules, such as CD40, CD80, CD86 and major histocompatibility complex (MHC) II and is also mediated by the production of some immune inhibitory factors, such as IL-10 and Tregs [19]. Tregs play a critical role in the maintenance of peripheral self-tolerance. They naturally express Foxp3, a transcription factor required for the establishment and maintenance of the Treg lineage identity and suppressor function. On one hand, Tregs influence all major subpopulations of APCs, including DCs, by the down-regulation of their antigen presenting function and the up-regulation of immunosuppressive cytokines. On the other hand, the functional state of DCs is important in determining Treg biology. The mutual interaction of DCs and Tregs may be crucial for the maintenance of peripheral tolerance [20]. To modulate DC function, GM-CSF has been used to induce semimature DCs that recruit Tregs, thereby preventing autoimmune thyroiditis, myasthenia gravis and type 1 diabetes in non-obese diabetic mice [21], [22], [23].

In addition, MDSCs isolated from spleen and bone marrow have been recently reported as a new mechanism for immunosuppression in a wide variety of unrelated pathologic conditions [24]. In mice, these cells are fairly well characterized as CD11b^+^Gr-1^+^. It has been demonstrated that large amounts of GM-CSF may be responsible for the expansion of the myeloid cell pool in secondary lymphoid organs, which in turn can recruit Tregs and thereby result in the suppressive effects on the immune response against tumors or infections [25], [26]. Thus, in the present study, we characterized the effect of the co-administration of a GM-CSF plasmid on the immune response induced by a JEV DNA vaccine expressing prM-E. We also investigated if GM-CSF affects the immune suppression by modulating the maturation status and function of DCs or inducing Tregs by a process that involves MDSCs. Our results indicate that co-inoculation of the JEV prM-E antigen with GM-CSF causes substantial dampening of the vaccine-induced immune responses and poor protection against lethal JEV challenge, and it is associated with the induction of immature DCs and the expansion of CD4^+^CD25^+^Foxp3^+^ Tregs but not MDSCs. Taken together, our findings not only provide valuable information for the clinical application of GM-CSF but also offer further insight into the understanding of the complex versatility of GM-CSF.

## Results

### Co-inoculation of the GM-CSF plasmid suppressed antibody (Ab) responses induced by the prM-E plasmid

DNA plasmids expressing premembrane and envelope (*prM-E*) genes of JEV, dengue virus serotype 1 (DENV1) and DENV2 were constructed in this study and named pCAG-JME, pCAG-D1ME and pCAG-D2ME, respectively. Similarly, DNA plasmids encoding the core (*C*) and envelope1 (*E1*) genes of the hepatitis C virus (HCV) were constructed and named pCAG-HCV-C and pCAG-HCV-E1, respectively. The plasmid encoding the murine GM-CSF fragment was named pCAG-GM. All plasmids were confirmed (data not shown) and purified for immunization.

**Figure 1:**
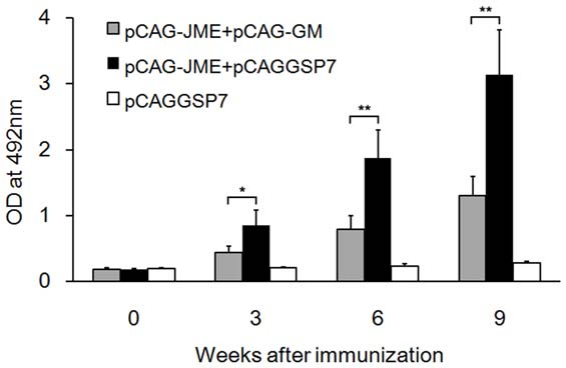
Dynamics of the Ab response of JEV DNA-immunized mice were detected by ELISA. Booster administrations were performed at week 3 and 6. Pre- and post-immunization serum samples (n = 10, 1∶800) were collected and then Ab titers were determined. The bar graph shows the mean ± standard deviation (SD) values for optical density (OD) of the group vaccinated with plasmids: gray-colored bars, mice co-inoculated with 50 µg pCAG-JME and 50 µg pCAG-GM; black bars, mice inoculated with a mixture of 50 µg pCAG-JME and 50 µg pCAGGSP7; hollow bars, mice inoculated with 100 µg pCAGGSP7 empty vector alone (*, *p*<0.05; **, *p*<0.01, one-way ANOVA test).

BALB/c mice were vaccinated with 100 µg plasmid by intramuscular (i.m.) injection three times at three-week intervals (0, 3 and 6). Mice sera were collected before or after immunizations to analyze the dynamics of the Ab response by enzyme-linked immunosorbent assay (ELISA) (Figure 1). As expected, the Ab levels, as measured by OD values, in the group inoculated with pCAG-JME and pCAGGSP7 were dramatically increased three weeks after the prime vaccination and were then greatly enhanced following the double booster immunization. However, Ab levels in mice co-administered with the *GM-CSF* gene were significantly lower than that of the pCAG-JME+pCAGGSP7 group, even following the second and third immunizations (*p*<0.01).

**Figure 2:**
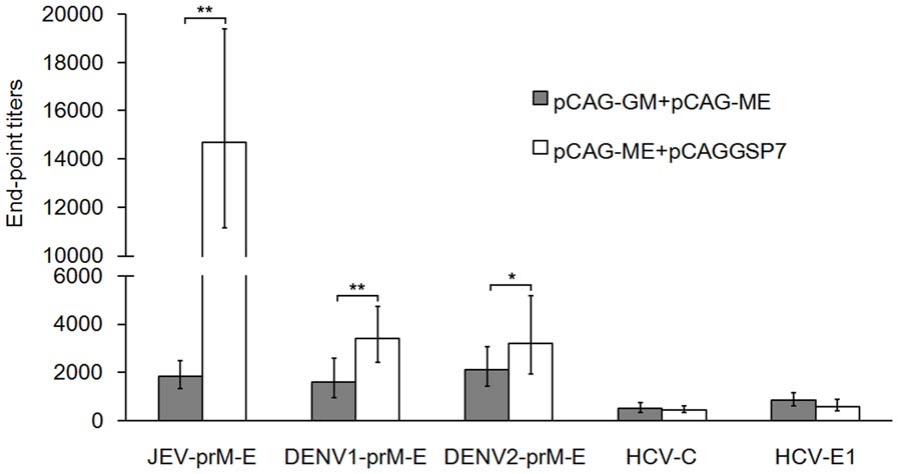
Co-inoculation of GM-CSF plasmid showed the influence on the vaccine-induced Ab responses. Sera were collected from immunized mice (n = 5) three weeks after the final vaccination. The end-point titers of anti-prM-E Abs were measured by ELISA and recorded as geometrical mean titers (GMT). Gray bars, mice co-inoculated with 50 µg pCAG-GM and 50 µg pCAG-JME (JEV), pCAG-D1ME (DENV1), pCAG-D2ME (DENV2), pCAG-HCV-C (HCV-C) or pCAG-HCV-E1 (HCV-E1); hollow bars, mice inoculated with a mixture of 50 µg pCAG-JME, pCAG-D1ME, pCAG-D2ME and 50 µg pCAGGSP7 (*, *p*<0.05; **, *p*<0.01, t test).

Meanwhile, serum samples obtained three weeks after the final immunization were also measured for end-point titers by anti-JEV IgG ELISA (Figure 2). Mice receiving the pCAG-JME and pCAGGSP7 plasmids showed high anti-JEV levels, with a geometrical mean titer (GMT) of approximately 1∶14,700. However, co-immunization of pCAG-GM with pCAG-JME showed an inhibitory effect on specific Ab production, with a low GMT of only up to 1∶1838. There was a significant difference between the pCAG-JME+pCAGGSP7 group and the pCAG-JME+pCAG-GM group (*p*<0.01).

Moreover, to investigate if the immunosuppressive effect of GM-CSF was related to antigens expressed by co-immunized DNA vaccines, immunogens from other viruses in the *Flaviviridae* family were used to co-immunize mice with pCAG-GM, and serum samples were also evaluated for end-point titers by anti-DENV or anti-HCV IgG ELISA (Figure 2). The data showed that mice receiving the pCAG-D1ME or pCAG-D2ME and pCAGGSP7 plasmids had similar specific Ab levels, with GMTs of approximately 1∶3200. In contrast, mice co-inoculated with pCAG-GM and pCAG-D1ME or pCAG-D2ME induced low Ab levels, with GMTs of only up to 1∶1600 (*p*<0.01) and 1∶2111 (*p*<0.05). Surprisingly, in contrast to above results, co-inoculation with pCAG-GM showed an enhancing trend of the immune responses induced by pCAG-HCV-C or pCAG-HCV-E1, with GMTs of 1∶ 527 (HCV-C) or 1∶857 (HCV-E1), which were slightly higher than the GMTs (1∶ 460 for HCV-C or 606 for HCV-E1) in groups co-inoculated with pCAGGSP7. These results indicated that the suppressive effect of GM-CSF on the vaccine-induced immune response with the co-inoculation was likely related to the immunogens used in immunization, at least for the *Flaviviridae* family.

**Figure 3:**
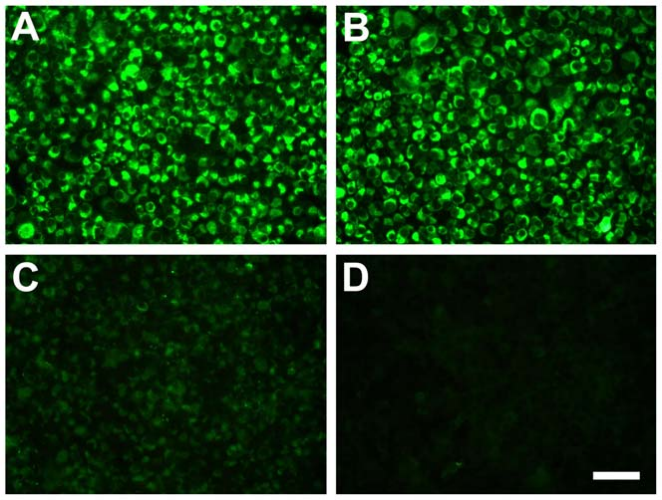
JEV-infected Vero cells reacted with DNA-immunized mice sera and visualized by IFA. Mice sera were obtained three weeks after the final immunization. (A) Serum from mouse immunized with pCAG-JME+pCAGGSP7. (B) Mouse anti-JEV E glycoprotein mAb as a positive control. (C) Serum from mouse immunized with pCAG-JME+pCAG-GM. (D) Serum from mouse immunized with pCAGGSP7. The figures shown are representative of five independent experiments performed. The scale bar is 50 µm.

To further evaluate the strength of the immune responses in mice immunized with different DNA plasmids, anti-JEV Ab levels were evaluated by indirect immunofluorescence assay (IFA) (Figure 3). Intense fluorescence in the cytoplasm was observed in the pCAG-JME+pCAGGSP7 group (Figure 3A), and it was similar to that of the positive control (Figure 3B), in which the cells were probed with JEV E glucoprotein monoclonal Ab (mAb). However, the pCAG-JME+pCAG-GM group showed very weak fluorescence in the cytoplasm (Figure 3C). Serum from pCAGGSP7-immunized (Figure 3D) mice failed to show any specific fluorescence. Consistent with the results of the ELISA, the pCAG-JME+pCAGGSP7 group induced an effective Ab response, whereas pCAG-JME+pCAG-GM did not, further indicating that co-inoculation of GM-CSF suppressed the immune response induced by pCAG-JME.

**Figure 4:**
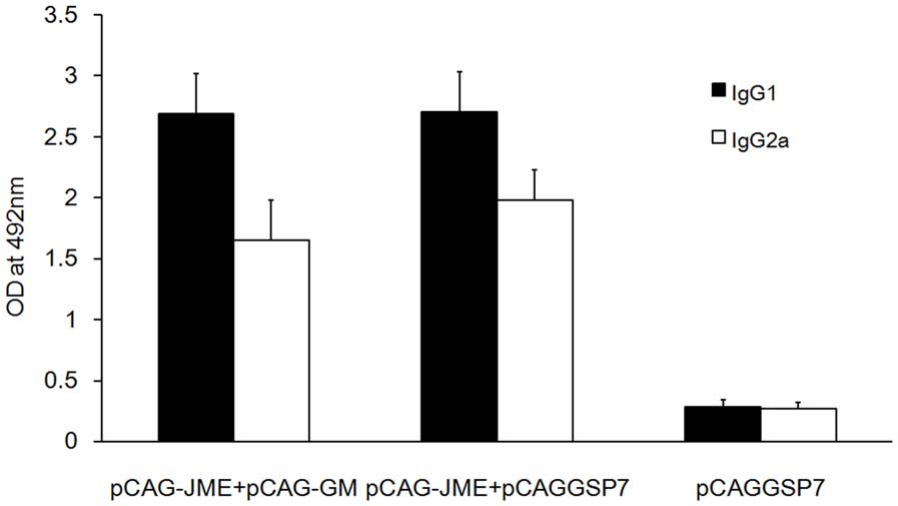
Mouse-specific serum IgG subclass responses to JEV DNA immunization were determined by ELISA. Sera (1∶200) were collected from vaccinated mice (n = 10) three weeks post-immunization. Values reported above for each group are the mean ± SD of the OD at 492 nm. The solid bars represent the IgG1 subtype, and the hollow bars represent the IgG2a subtype. The IgG2a/IgG1 ratios of three groups are 0.613±0.045, 0.734±0.057 and 0.954±0.062, respectively.

Serum levels of the JEV-specific IgG isotypes were also determined to further assess the efficacy of the DNA vaccine and the *GM-CSF* gene in the induction of Th1- or Th2-like immune responses. The amount of JEV-specific IgG1 and IgG2a subtypes were markedly augmented in the groups with or without pCAG-GM, compared to the control group (pCAGGSP7 alone) (Figure 4). Further, the IgG2a/IgG1 ratio in the control group was approximately 1, whereas the ratios in groups of immunized mice either with or without pCAG-GM were decreased, with ratios of 0.613±0.045 and 0.734±0.057, respectively, indicating the participation of Th2 cells in the response to JEV prM-E proteins. However, there was no statistic difference between the IgG2a/IgG1 ratios of the pCAG-JME+pCAG-GM group and the pCAG-JME+pCAGGSP7 group, suggesting that the balance of Th1 and Th2-associated immunity might be not affected by the co-injection of GM-CSF.

### The protective immunity elicited by the JEV prM-E DNA vaccine was diminished by adjuvant treatment with the GM-CSF plasmid

**Figure 5:**
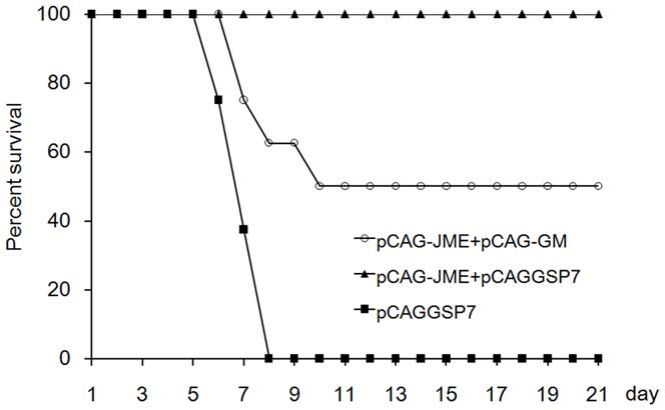
Protective immunity elicited by JEV-prM-E DNA vaccines. Mice (n = 8) were challenged with a dose of 50 LD50 of JEV (Beijing-1) three weeks post-immunization followed by daily monitoring for 21 days, and the percentage of survivors was calculated (*p*<0.05, pCAG-JME+pCAG-GM group vs. pCAG-JME+pCAGGSP7 group, log-rank test).

BALB/c mice were vaccinated with plasmids three times and intraperitoneally challenged three weeks post-immunization with 50 LD50 of the JEV Beijing-1 strain. As shown in Figure 5, all mice in the pCAG-JME+pCAGGSP7 group survived the JEV challenge, whereas mice co-immunized with the GM-CSF plasmid were not fully protected, with a 50% survival rate (four of eight, *p*<0.05), which is consistent with the low JEV-specific IgG level. Mice immunized with pCAGGSP7 alone were not protected (negative control). This result demonstrated that using the murine *GM-CSF* gene as an adjuvant to JEV prM-E DNA immunization hampered the ability of the vaccine to expand the protective immunity.

### The suppressive effect of pCAG-GM was dose-dependent

**Figure 6:**
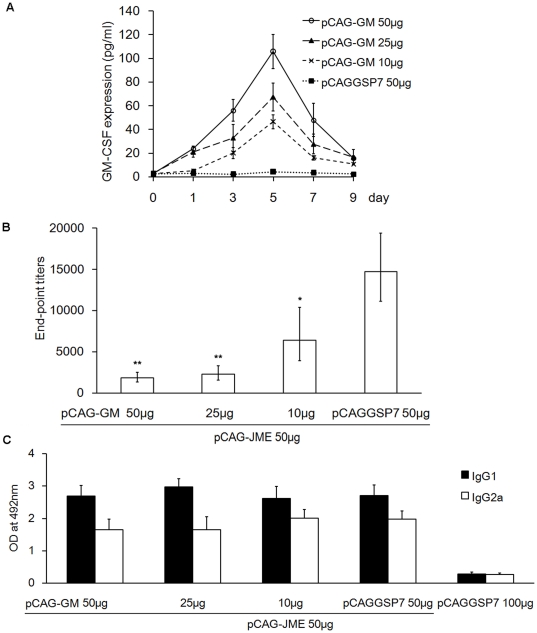
The suppression of Ab responses by the GM-CSF plasmid was dose-dependent. (A) Mice (n = 5) were inoculated with various dosages of pCAG-GM (10, 25 and 50 µg) or pCAGGSP7 (50 µg) without booster and the expression levels of GM-CSF in the undiluted sera were monitored by ELISA at pre-inoculation and 1, 3, 5, 7 and 9 day(s) post-inoculation. The expression levels of GM-CSF are shown as the mean concentration with a SD (*p*<0.01, one-way ANOVA test). (B, C) Mice (n = 5) were immunized with pCAG-JME (50 µg) plus various doses of pCAG-GM (10, 25 and 50 µg) or pCAGGSP7 (50 µg) three times at three-week intervals. Mice treated with 100 µg pCAGGSP7 served as the control. Three weeks after the final immunization, serum samples were collected and the Ab immune response was detected by ELISA. The levels of specific anti-JEV-prME Abs are shown as GMT (*, *p*<0.05; **, *p*<0.01, vs. pCAG-JME 50 µg+pCAGGSP7 50 µg group, one-way ANOVA test) (B). The levels of JEV-specific serum (1∶200) IgG subclasses are shown as the OD value (C), and the solid bars represent the IgG1 subtype, the hollow bars represent the IgG2a subtype. The IgG2a/IgG1 ratios of five groups are 0.613±0.045, 0.554±0.041, 0.765±0.055, 0.734±0.057 and 0.954±0.062, in turn.

To determine if the suppressive effect of GM-CSF was related to its expressed amount, mice were inoculated with various doses of pCAG-GM (10, 25 and 50 µg) or pCAGGSP7 (50 µg) without booster and then the expression level of GM-CSF in the serum was monitored by ELISA at different time points as indicated. As shown in Figure 6A, expression of GM-CSF gradually increased, the peak value was seen on day 5 after inoculation and then it decreased during observed period. The highest concentrations of GM-CSF in 10, 25 and 50 µg groups were 46.46±5.88, 67.36±11.81 and 105.84±14.58 pg/ml respectively, and they showed significant difference (*p*<0.01). This indicated that the expression levels of GM-CSF were closely associated with the inoculated amounts. To investigate if the suppressive effect of GM-CSF was dose-dependent, groups of mice were immunized with 50 µg pCAG-JME plasmid plus various doses of pCAG-GM (10, 25 and 50 µg) or pCAGGSP7 (50 µg), followed by two boosters at three-week intervals. The end-point titers were measured three weeks after the final immunization. Animals treated with 100 µg pCAGGSP7 served as a control. As shown in Figure 6B, co-administration of 25 and 50 µg pCAG-GM resulted in a significant reduction of specific JEV Ab titers (*p*<0.01), whereas mice co-inoculated with 10 µg pCAG-GM had less of an effect on anti-prM-E Ab responses (*p*<0.05), indicating that the suppressive effect of GM-CSF was dose-dependent. Meanwhile, the levels of the IgG1 and IgG2a subtypes were also measured by ELISA. The results showed that co-administration with high doses of pCAG-GM (25 and 50 µg) generated lower levels of IgG2a than IgG1 and a lower IgG2a/IgG1 ratio with 0.554±0.041 and 0.613±0.045, respectively, compared with that (0.765±0.055) of the low dose (10 µg) group (Figure 6C). However, there was no significant difference in IgG2a levels between high doses (25 and 50 µg) groups and lower dose (10 µg) group.

### The suppressive effect of pCAG-GM was timing-dependent

**Figure 7:**
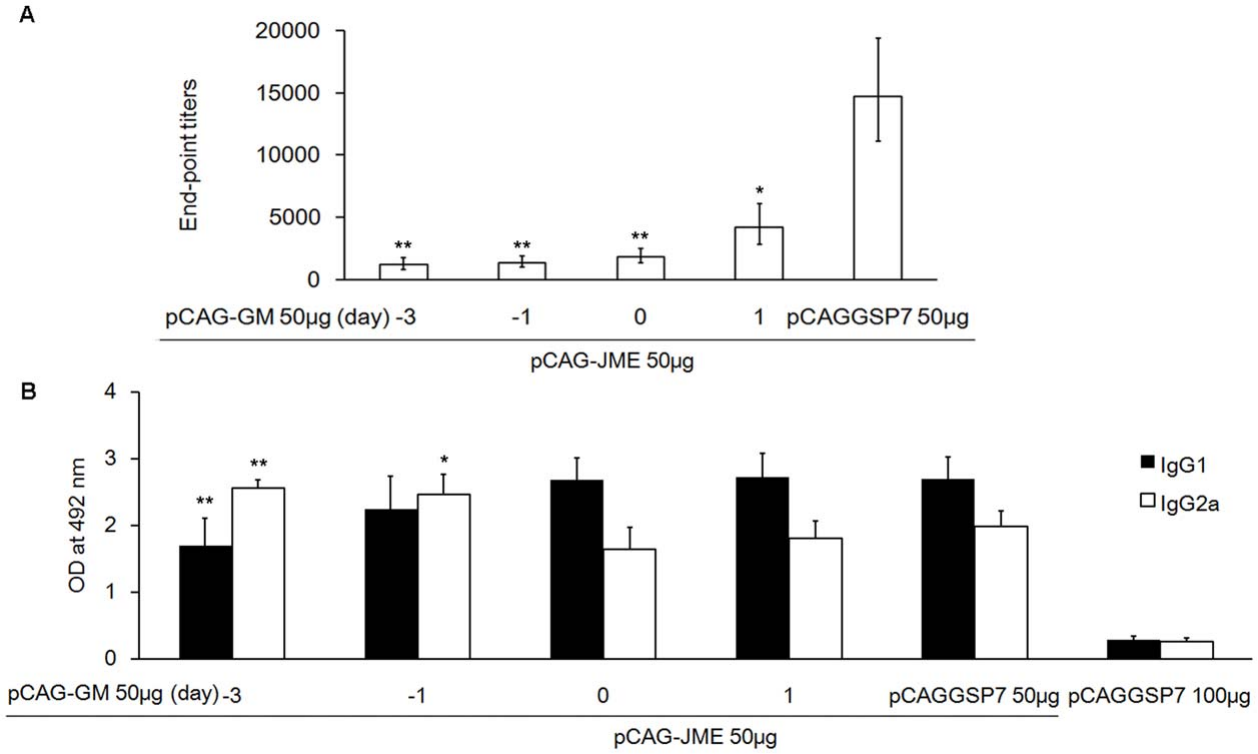
The suppressive effect of the GM-CSF plasmid on Ab responses was timing-dependent. Mice (n = 5) were vaccinated with 50 µg pCAG-JME with 50 µg pCAG-GM or pCAGGSP7. The numbers beneath the x-axis indicate the timing of immunization. The 0 indicates that the GM-CSF plasmid was given with pCAG-JME simultaneously, −3 or −1 indicates that the plasmid was given 3 days or 1 day ahead of pCAG-JME delivery, respectively, and 1 indicates that pCAG-GM was given 1 day after pCAG-JME vaccination. Mice treated with 100 µg pCAGGSP7 served as the control. All animals received two booster doses at three-week intervals. Three weeks after the final immunization, serum samples were collected and the Ab immune response was detected by ELISA. The levels of specific anti-JEV-prME Abs are shown as GMT (A). (*, *p*<0.05; **, *p*<0.01, vs. pCAG-JME 50 µg+pCAGGSP7 50 µg group, one-way ANOVA test). The levels of JEV-specific serum (1∶200) IgG subclasses are shown as the OD value (B), and the solid bars represent the IgG1 subtype, the hollow bars represent the IgG2a subtype (*, *p*<0.05; **, *p*<0.01, vs. 0d, 1d or pCAGGSP7 50 µg groups; one-way ANOVA test). The IgG2a/IgG1 ratios of these six groups are 1.509±0.141, 1.094±0.099, 0.613±0.045, 0.662±0.065, 0.734±0.057 and 0.954±0.062, in turn.

To determine if the suppressive effect of GM-CSF was associated with the timing of the pCAG-GM injection, BALB/c mice were inoculated with 50 µg pCAG-JME+50 µg pCAG-GM or pCAGGSP7 at the same time, or with pCAG-GM 1 or 3 day(s) ahead or 1 day after pCAG-JME inoculation. Three weeks following the three immunizations, the levels of anti-JEV prM-E Ab in the sera were analyzed by ELISA. As shown in Figure 7A, co-administration of pCAG-GM before, together with or after pCAG-JME delivery significantly suppressed the anti-JEV prM-E Ab titers (*p*<0.05). Mice receiving pCAG-GM 1 or 3 day(s) before pCAG-JME delivery had slightly lower anti-JEV titers than that in mice receiving pCAG-GM coincident with pCAG-JME. However, when pCAG-GM was given 1 day after pCAG-JME delivery, the Ab titers increased more than 2-fold compared to those obtained with giving pCAG-GM 1 or 3 day(s) ahead of pCAG-JME inoculation. This result indicated that pretreatment with pCAG-GM led to greater effects on the immune response induced by pCAG-JME.

Analysis of the anti-JEV Ab isotypes showed that inoculation with pCAG-GM 3 days before pCAG-JME immunization resulted in high IgG2a titers, indicating a primary Th1-biased response (Figure 7B). Injection of pCAG-GM 1 day prior to pCAG-JME delivery induced both a Th1 and a Th2 response. When pCAG-GM was inoculated simultaneously with or 1 day following the JEV DNA vaccination, the ratio of IgG2a/IgG1 gradually recovered to that of the pCAG-JME+pCAGGSP7 group, indicating an enhancement of Th2 immunity. The IgG1 and IgG2a levels in group pCAG-GM 3 days, and the IgG2a level in group pCAG-GM 1 day before pCAG-JME immunization were significantly different from that of other groups (simultaneously with, 1 day later and pCAG-JME+pCAGGSP7). The IgG2a/IgG1 ratios of these groups were 1.509±0.141, 1.094±0.099, 0.613±0.045, 0.662±0.065 and 0.734±0.057, respectively. These results suggested that the timing of GM-CSF co-administration significantly altered the subtype of the resulting Th response. Pretreatment with the pCAG-GM adjuvant indeed favored a shift to Th1 over Th2.

### Co-inoculation of pCAG-GM modulated cytokine production

**Figure 8:**
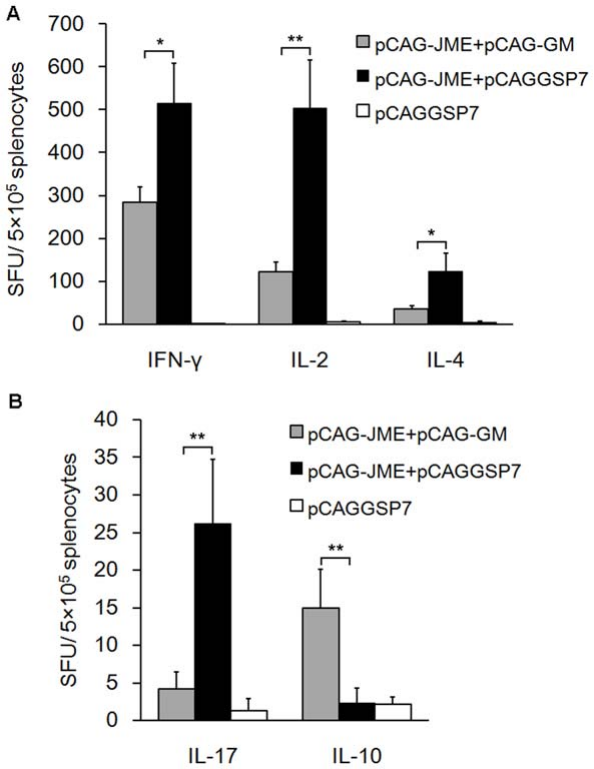
Analysis of the effect of pCAG-GM on splenocyte-secreted cytokines. Splenocytes were isolated from mice (n = 6) three weeks after the final immunization. The levels of cell-produced IFN-γ, IL-2 and IL-4 (A), IL-17 and IL-10 (B) following stimulation by concentrated JEV proteins for 48 h were measured by ELISPOT assays. The numbers of cytokine-positive cells are expressed as spot-forming units (SFU)/5×10^5^ cells after background subtraction (*, *p*<0.05; **, *p*<0.01, one-way ANOVA test).

The levels of IFN-γ, IL-2, IL-4, IL-17 and IL-10 cytokines secreted by splenocytes of mice immunized with plasmid DNA upon stimulation with the JEV antigen were examined by enzyme-linked immunospot (ELISPOT) assay. The results showed that the levels of IFN-γ, IL-2, IL-4 and IL-17 in the pCAG-JME+pCAGGSP7 group were significantly higher than those in the vector control group (Figure 8). However, the GM-CSF adjuvant dramatically decreased the secreted levels of IFN-γ (*p*<0.05), IL-2 (*p*<0.01), IL-4 (*p*<0.05) and IL-17 (*p*<0.01) when compared with the mice received pCAG-JME without pCAG-GM. Notably, in contrast, IL-10 production was significantly elevated in the pCAG-JME+pCAG-GM group (*p*<0.01) compared with the pCAG-JME+pCAGGSP7 and vector control groups. Because IL-2 and IFN-γ are markers of the Th1 response, IL-4 expression is used as a marker of the Th2 response, and IL17 is defined as a predominant marker of the Th17 pathway [27], these results indicated that the Th1-, Th2- and Th17-like immune responses were all stimulated by i.m. administration of the JEV prM-E DNA vaccine, whereas they were significantly inhibited by the pCAG-GM adjuvant. Interestingly, a dramatically higher level of IL-10, a inhibitory cytokine, was observed in the mice co-treated with pCAG-GM, which may suggest that the suppressive effect by pCAG-GM may be associated with the degree of DC maturation and/or generation of Tregs, as semimature DCs are involved in the immunogenic tolerance caused by GM-CSF via an increase in the induction of IL-10-producing Tregs [18], [28].

### Co-administration of pCAG-GM influenced DC maturation and induced the generation of Tregs but not MDSCs

**Figure 9:**
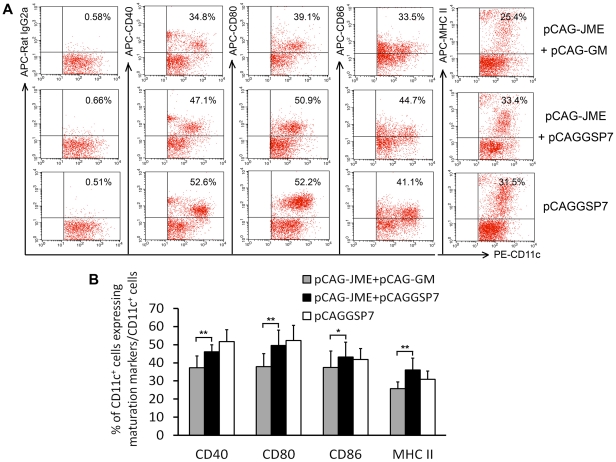
Analysis of the suppressive effect of pCAG-GM on DC maturation. 100 µl peripheral blood from immunized mice (n = 6) was stained with fluorescently conjugated mouse mAb to detect the expression of the surface markers CD11c, CD40, CD80, CD83 and MHC II on DCs. (A) Representative data from the FACS analysis of peripheral blood DCs from the immunized mice on the 8th day after the final vaccination. The percentage of double-positive cells is indicated in the top of right corner. (B) The bar graph shows the mean percentage with a SD of CD11c^+^ cells expressing maturation markers in different groups (*, *p*<0.05; **, *p*<0.01; one-way ANOVA test).

To investigate if the characteristics of immature DCs were altered by GM-CSF plasmid co-inoculation, the levels of costimulatory and antigen presentation-associated molecules, including CD40, CD80, CD86 and MHC II, of DCs in peripheral blood were evaluated by fluorescence-activated cell sorting (FACS). As shown in Figure 9, the expression levels of maturation surface markers of DCs were similar in the pCAG-JME+pCAGGSP7 and the vector control groups, but the markers were markedly down-regulated in the group co-immunized with pCAG-GM. This result indicated that the JEV prM-E DNA vaccine did not accelerate the maturation of DCs, whereas the co-injection of pCAG-GM significantly inhibited the maturation process of DCs.

**Figure 10:**
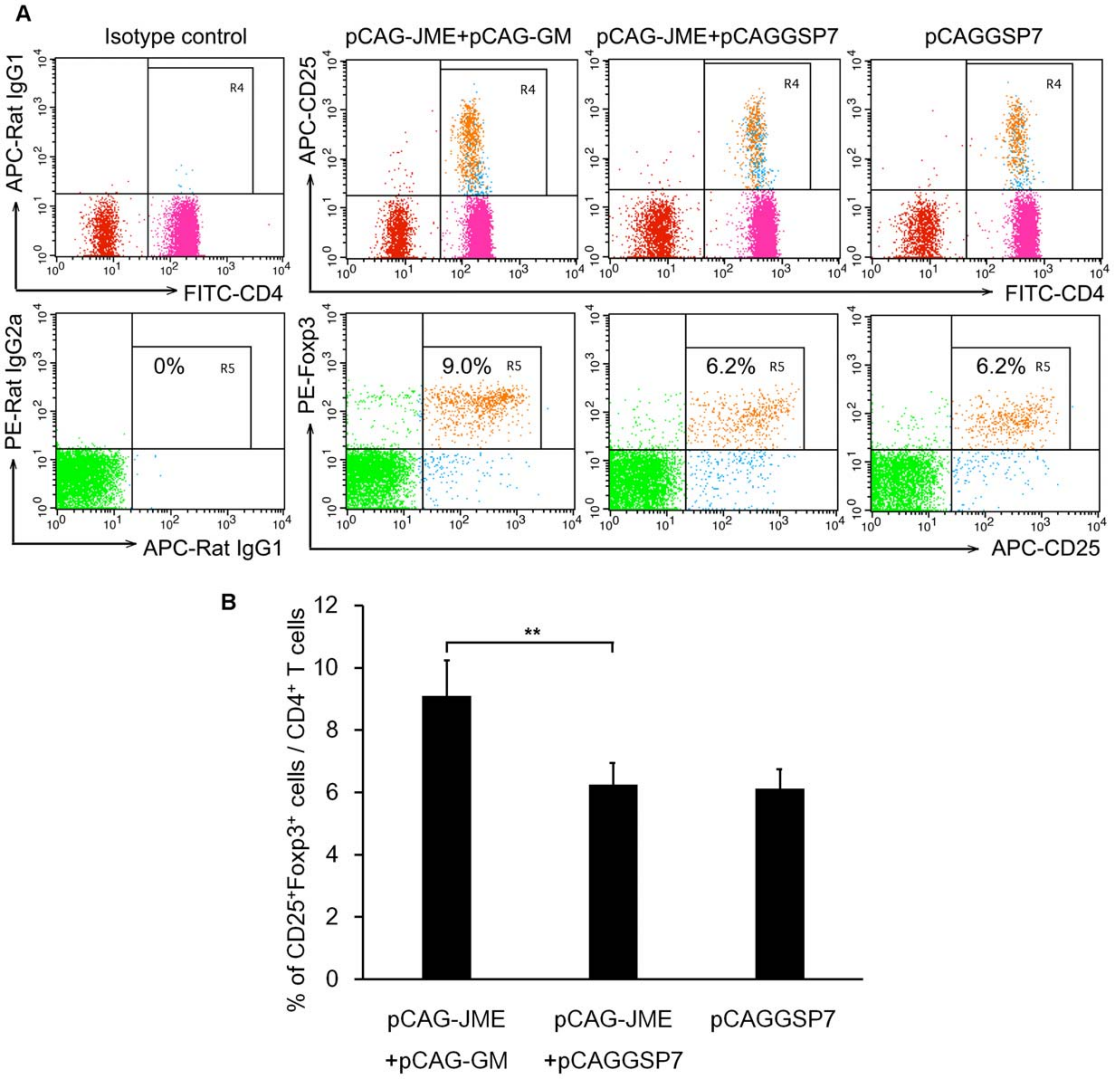
Analysis of the amplifying effect of pCAG-GM on Tregs. 100 µl peripheral blood from immunized mice (n = 6) was stained with fluorescently conjugated mouse mAbs to detect the surface expression of CD3e, CD4 and CD25 and the intracellular expression of Foxp3. (A) Representative data from the FACS analysis of peripheral blood CD4^+^CD25^+^Foxp3^+^ Tregs from the immunized mice three weeks after the final vaccination. The percentage of double-positive cells is indicated in the top of right corner. (B) The bar graph shows the mean percentage with a SD of CD4^+^CD25^+^ Foxp3^+^ Tregs among CD4^+^ T cells in different groups (**, *p*<0.01, one-way ANOVA test).

To examine if co-inoculation with the GM-CSF plasmid affected the relative numbers of Tregs, the surface expression of CD3e, CD4 and CD25 and the intracellular expression of the most accepted marker of Tregs, Foxp3, were detected in mouse peripheral blood cells by FACS. As shown in Figure 10, a significant increase in the percentage of CD3e^+^ CD4^+^ CD25^+^ Foxp3^+^ Tregs was observed in the group co-immunized with pCAG-GM and pCAG-JME when compared to the pCAG-JME+pCAGGSP7 and vector control groups. This result was consistent with the FACS analysis for the maturation of DCs, indicating that the low expression of the major costimulatory molecules of DCs might induce the generation of Tregs, turn off activated T cells and result in immune tolerance [20].

To further investigate the role of MDSCs in the immune tolerance induced by the GM-CSF plasmid, the surface expression levels of mouse CD11b and Gr-1 on peripheral blood cells were analyzed by FACS. Unfortunately, there was no obvious difference in the percentages of CD11b^+^ Gr-1^+^ MDSCs (data not shown) in pCAG-JME-immunized mice with or without pCAG-GM. This result demonstrated that MDSCs were not involved in the induction of the immune suppression by the GM-CSF plasmid under the experimental conditions in this study, although CD11b^+^ Gr-1^+^ MDSCs have a close relationship with immature DCs and Tregs.

### The GM-CSF plasmid adjuvant did not affect the immunogen expression of plasmid DNA vaccine

**Figure 11:**
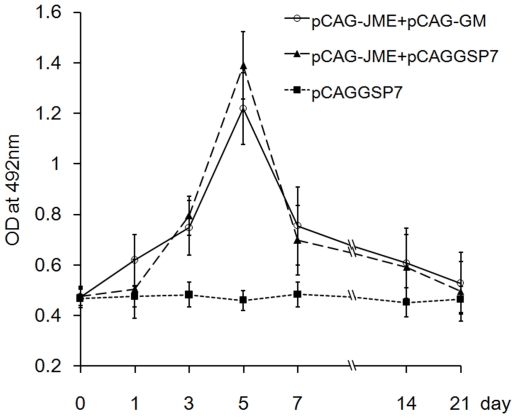
Kinetics of the expression of the JEV DNA vaccine immunogen. Mice (n = 5) were immunized with plasmids without booster. JEV prM-E protein expression was measured by ELISA in the undiluted sera collected at pre-inoculation and 1, 3, 5, 7, 14 and 21 days post-inoculation. Data are expressed as the mean values of OD with a SD.

To evaluate if co-inoculation with pCAG-GM affected the immunogen expression of the JEV DNA vaccine, the sera were collected at pre-inoculation and at 1, 3, 5, 7, 14 and 21 day(s) post-inoculation, and the level of JEV prM-E protein in the sera was measured by ELISA. As shown in Figure 11, the mice immunized with pCAG-JME either with or without pCAG-GM had significantly increased amounts of JEV prM-E protein. The elevated level of prM-E expression was observed as early as 3 days following vaccine inoculation and reached a peak on day 5, followed by a decrease. Expression was detected until day 14 following vaccination. No obvious differences were observed between these two groups. This result suggested that the GM-CSF plasmid adjuvant did not affect the immunogen expression of the DNA vaccine plasmid in this study.

## Discussion

### Co-inoculation of the GM-CSF plasmid depressed both the humoral and cellular immune responses induced by JEV DNA vaccines expressing prM-E

As a potential cytokine adjuvant of DNA vaccines, GM-CSF has received considerable attention for its essential role in the recruitment of APCs and the differentiation, growth and maturation of DCs. In addition, as a well-recognized regulator of hematopoiesis, GM-CSF is commonly administered in clinical practice to treat neutropenia and enhance leukocyte activity. However, our recent study of a JEV DNA vaccine [10] showed that co-inoculation of the GM-CSF plasmid significantly suppressed the specific IgG response and resulted in decreased protection against JEV challenge. These data raise the concern that the use of GM-CSF as an adjuvant or as a treatment agent might be harmful because GM-CSF can impair the vaccine-induced or anti-tumor immune responses under conditions that remain unclear. In this study, we first confirmed the suppressive effect of the co-administration of a GM-CSF plasmid on the immune response induced by a JEV DNA vaccine and the properties of this suppression. It was found that the plasmid expressing the JEV protein prM-E could elicit high levels of JEV-specific Abs, with a GMT of 1∶14,700, and a 100% survival rate in animals (Figures 1, 2 and 5), whereas mice co-inoculated with the GM-CSF plasmid and the JEV prM-E plasmid had a low level of immune response, with a low end-point Ab titer of 1∶1838, and a low protective rate (50%). This result indicated that the co-immunization of the GM-CSF plasmid did not enhance the vaccine-induced specific Ab response, nor did it provide sufficient protective immunity against JEV challenge.

The effects of the GM-CSF plasmid on the cellular immune response were also investigated in this study, and the levels of IFN-γ, IL-2, IL-4, IL-17 and IL-10 cytokines secreted by splenocytes from immunized mice were examined by ELISPOT. High levels of IFN-γ, IL-2, IL-4 and IL-17 were observed in the pCAG-JME+pCAGGSP7 group, whereas the GM-CSF plasmid adjuvant markedly inhibited the secreted levels of IFN-γ, IL-2, IL-4, and IL-17 (Figure 8). These cytokines represent the major factors involved in various T cell subsets and different types of immune responses. Therefore, the results indicated that the pCAG-GM adjuvant significantly inhibited all the Th1-, Th2- and Th17-like immune responses elicited by the DNA vaccine.

It is noteworthy that a significantly elevated level of IL-10 was observed in the pCAG-GM co-treated mice in this study (Figure 8B). This was consistent with a study of the suppressive effect of GM-CSF on the progress of autoimmune disease, in which IL-10 levels were significantly increased in GM-CSF-treated mice, and IL-10 was shown to be essential for disease suppression in these animals [28]. It is known that IL-10 is not only a marker of the Th2 response but also acts as an inhibitory cytokine involved in the modulation of DC maturation and the generation of Tregs [29]. Accordingly, in our study, IL-10 may be associated with immunogenic tolerance caused by GM-CSF via the increased induction of Tregs and immature DCs (see below).

Furthermore, JEV-specific IgG isotype Ab levels were also determined, and the control group mice had similar levels of IgG2a and IgG1 in the sera, whereas reduced IgG2a/IgG1 ratios were observed in both groups of immunized mice with or without pCAG-GM (Figure 4). Because IgG2a and IgG1 represent Th1 and Th2 immune cell types, respectively, this finding indicated a bias toward the Th2 immune response to JEV prM-E proteins. However, the use of the GM-CSF adjuvant caused an insignificant change in IgG1 level and a little decreased level of IgG2a. Consistent with the results of cytokines and specific IgG titers, this result suggested that co-injection of the GM-CSF plasmid could suppress both the humoral and cellular immune responses induced by JEV prM-E DNA vaccines.

In agreement with our findings, another study of an JEV DNA vaccine [30] also demonstrated that the co-administration of an IL-12-expressing plasmid could be detrimental to the immune responses elicited by the DNA vaccine encoding the envelope (E) protein of JEV. Similarly, in another study of an HIV-1 DNA vaccine [11], the GM-CSF-encoding plasmid was reported to fail to augment the immune responses induced by a plasmid encoding HIV-1 gp120, and an elevated level of type I IFN in the local site of inoculation was associated with the inhibitory effect of the GM-CSF plasmid in vivo. Recently, opposite immune functions of GM-CSF administered as a vaccine adjuvant in cancer patients was reported [31]. Moreover, GM-CSF has been used to effectively induce immune tolerance in animal models of experimental autoimmune disease, including myasthenia gravis and diabetes, and the beneficial effect of GM-CSF therapy in these diseases was shown to be mediated through the promotion of tolerogenic DCs and the expansion of Tregs [22], [23].

However, our results were not consistent with some reports of DNA vaccine studies in which GM-CSF acted as the adjuvant and could enhance immune responses via several mechanisms, including the activation of granulocytes, macrophages and natural killer T cells and the promotion of the local recruitment and maturation of DCs, which likely led to the improvement of tumor antigen presentation to T lymphocytes [32], [33], [34].

These reports demonstrate contrasting GM-CSF-induced effects: on one hand, because GM-CSF plays important roles in the enhancement of immune responses, it has been recommended as a cytokine adjuvant in some vaccines and as a treatment to “boost” the immune response in infection or cancer patients. On the other hand, GM-CSF can induce immune tolerance and has been used to treat autoimmune diseases. Although we cannot completely explain this apparent paradox, it is thought to be related to several factors, including the immunogen, the dose, which may be an important determinant, and the timing of GM-CSF administration. Further studies are needed to investigate the mechanisms underlying the suppressive phenomenon and how GM-CSF can be used safely in clinic.

### The immune suppression of the GM-CSF plasmid was dose- and timing-dependent and could be related to the immunogenicity of the antigens

The suppressive effect of GM-CSF has been considered to be associated with the doses of GM-CSF used in the study, and a large amount of GM-CSF might cause immune suppression. To confirm this, we used different doses of pCAG-GM to co-immunize mice. Unsurprisingly, it was demonstrated that the expressed levels of GM-CSF were closely related to the inoculated amounts and the suppressive effect weakened with decreasing doses of GM-CSF plasmid (from 10 to 50 µg), and the highest dose of pCAG-GM (50 µg) resulted in the lowest specific JEV Ab titer (Figure 6), suggesting that suppressive effect of GM-CSF was dose-dependent.

Recent studies reported by Parmiani *et al* [31] concluded that the dose of GM-CSF used as an immune adjuvant was paramount in clinical trials. Relatively lower doses (40–80 µg for 1–5 days in vaccinated cancer patients) could elicit an immune response, whereas higher doses (100–500 µg) showed no advantage, even lost its efficacy and involved in its clinical implications resulted in immunosuppression *in vivo* under certain circumstances. In other words, high dose of GM-CSF was likely associated with side effects and immunotoxicity. Serafini *et al* [35] reported that the expressive level of GM-CSF at 58 pg/ml in mice sera was low enough in enhancing the immune response induced by the tumor vaccine, while the serum level of GM-CSF at 206 pg/ml showed an inhibitory role. However, in our study, the peak serum concentration of GM-CSF from the lowest dose (10 µg) group was 46.46 pg/ml, it still caused a markedly suppressive effect in JEV DNA vaccine. Thus we proposed that the expressed level of GM-CSF with lowest dose used in this study was likely to exceed the maximum range required for enhancement of the JEV DNA vaccine. Taken together, the dose of GM-CSF was thought to be a crucial factor in determining the strength and state of vaccine-induced immune responses but not the unique one and other factors such as experimental condition, immunogen and administration timing were also involved in.

Moreover, to determine if immune suppression of GM-CSF was associated with the timing of the treatment, pCAG-GM was inoculated prior to or after pCAG-JEM vaccination. Mice injected with pCAG-GM before pCAG-JME delivery had a strong inhibition of Ab titers compared with mice in which pCAG-GM was simultaneously given with pCAG-JME. When pCAG-GM was given 1 day later, the inhibitory effect of GM-CSF was slightly weaker, and the anti-JEV Ab titer showed an increased trend but was still markedly lower than that in the group given pCAG-JME+pCAGGS7 (Figure 7). This result implied that the pretreatment with GM-CSF had significant effects on the vaccine-induced immune response. Delaying the addition of GM-CSF resulted in progressively less inhibition. Interestingly, changes in the IgG2a/IgG1 ratio seemed to be closely associated with the time of delivery, and the inoculation with pCAG-GM 3 days before pCAG-JME delivery resulted in a high IgG2a titer and high IgG2a/IgG1 ratio. The strength of the IgG2a and IgG1 responses was similar when pCAG-GM was injected 1 day prior to pCAG-JME delivery. High IgG1 titers and the IgG2a/IgG1 ratio gradually recovered to that of the pCAG-JME+pCAGGSP7 group when pCAG-GM was given simultaneously with or 1 day after the pCAG-JME vaccination. These results suggested that the pretreatment with pCAG-GM elicited a response that was primarily biased towards Th1, whereas simultaneous co-inoculation or post-treatment enhanced Th2 immunity. This was not consistent with another study [36], in which pretreatment with the GM-CSF plasmid primarily elicited a Th2 response and the simultaneous injection of the GM-CSF plasmid with the DNA vaccine activated both a Th1 and a Th2 response. When GM-CSF was administered 3 days after DNA vaccination, there was a predominant enhancement of Th1 immunity. We cannot explain this contradiction by our results, and it may be related to the experimental conditions. Nevertheless, our results and those of others suggested that the timing of GM-CSF co-administration also influenced immune responses and markedly altered the phenotype of the resultant Th response.

In addition, to investigate if the immune suppression of pCAG-GM was related to the JEV prM-E protein, pCAG-GM was also co-administered with pCAG-D1ME, pCAG-D2ME, pCAG-HCV-C or pCAG-HCV-E1. Interestingly, a slight inhibitory effect on the Ab responses induced by the DENV DNA vaccines was observed, whereas pCAG-GM co-inoculation showed an enhancing trend in HCV-C- or HCV-E1-induced immune responses (Figure 2). We cannot explain why GM-CSF played opposite roles in the immune responses induced by different vaccines, but the diverse immunogenicity of these antigens was thought as one of the key reasons. It is known that both the prM-E proteins of DENVs, JEV and C, E1 proteins of HCV have different immunogenicities and that the properties of the immune responses they induce are different. For example, the prM-E protein of JEV is a strong immunogen, and JEV infection predominantly induces a humoral immune response, whereas DENV and HCV infections predominantly induce cellular immune responses, although Ab reactions are also involved in the immune responses. As known, for various antigen peptides or epitopes with different immunogenicity, the different antigen recognition and presentation might define the immunologic outcome. Moreover, served as the professional APC, DCs maturation was found to be influenced by the co-delivery of GM-CSF. Therefore, the different effects of GM-CSF in different but related vaccine antigens might be also associated with the modulation of antigen recognition and presentation pathways. Further studies are required for investigating which kind of antigen GM-CSF would enhance in the antigen presentation.

Together, the above results suggested that the immune suppression of plasmid GM-CSF was dose- and timing- dependent and may be closely related to the immunogenicity of the antigen, further indicating that the roles of GM-CSF have complex versatility. Therefore, caution should be exercised in the safe use of GM-CSF as an adjuvant in vaccination trials or as a therapeutic agent in cancer or transplantation patients. In further studies, it will be important to establish a dose range and time schedule of GM-CSF co-administration with different DNA vaccinations, i.e., to determine under which conditions GM-CSF is able to activate the immune system or harness the immoderate immune response or induce immune tolerance, in order to meet the demands of various clinical application.

### Immature DCs and Tregs but not MDSCs were involved in the immunosuppression induced by GM-CSF

Tolerance can be defined as the inability of a host to respond to antigens, and it can be generated centrally or peripherally. A number of mechanisms, including antigen expression, DC maturation, Tregs and MDSCs generation, may contribute to the tolerance or immune suppression caused by cytokine adjuvants. In our study, it was hypothesized that the suppression might be due to the reduction in JEV antigen expression caused by GM-CSF expression; therefore, serum levels of the JEV prM-E protein were determined by ELISA. However, there were no significant differences in the serum protein levels between the two groups of mice inoculated with or without pCAG-GM (Figure 11), indicating that the treatment with GM-CSF did not affect JEV prM-E expression and secretion; thus, the suppression could mainly result from other factors. DCs have been shown to contribute to T-cell tolerance, and the immature developmental stages of DC differentiation were believed to induce T-cell anergy or Tregs. whereas the DCs that can transform into mature DCs under exposure to activating stimuli were thought to be immunogenic, with the capability of promoting an effector T cell response [18], [20], [37]. Current evidence indicates that the immunogenic or tolerogenic function of DCs is largely determined by differentiation status, and GM-CSF plays an essential role in the promotion of the differentiation. Therefore, we investigated if there was enhancement of immature DCs in the blood after pCAG-GM co-inoculation. As expected, the expression of the mouse DC surface markers CD11c, CD40, CD80, CD83 and MHC II was drastically down-regulated in the group co-immunized with pCAG-GM (Figure 9), indicating that the co-injection of pCAG-GM significantly inhibited the maturation process of DCs.

Recent studies have shown that DCs may exert their tolerogenic functions through the generation of Tregs, and Foxp3 is currently the best available marker for these cells [37], [38]. Accordingly, a significant expansion in the population of CD4^+^CD25^+^Foxp3^+^Tregs was observed in mice co-inoculated with GM-CSF (Figure 10), strongly indicating a possibility that GM-CSF treatment mobilized Tregs via tolerogenic DCs. Moreover, we analyzed cytokine production by assessing their expression levels in splenic lymphocytes isolated from treated animals. GM-CSF-treated mice had an increased expression of IL-10 (Figure 8) but not TGF-β (data not shown). It is known that IL-10 and TGF-β are important inhibitory cytokines, and this result suggested that the immunosuppression induced by GM-CSF was likely related to IL-10 but not TGF-β. In fact, IL-10 can be secreted by many immune cells, including CD4^+^CD25^+^ Tregs and DCs, and the function of DCs, Tregs and IL-10 are interrelated. Immature DCs which can produce IL-10 and induce IL-10-producing Tregs are capable of inducing immunogenic tolerance [28], [29], [39], [40]. How these cells interact is not clear and will be the focus of further studies.

In addition, more recent studies have demonstrated that MDSCs act as a new mechanism of immune suppression and are characterized as CD11b^+^Gr-1^+^ in mice. It has been accepted that MDSC expansion and activation are likely controlled by GM-CSF, and high doses of GM-CSF-producing vaccines impair the immune response through the recruitment of MDSCs [41]. In this study, we also analyzed the percentage of CD11b^+^Gr-1^+^ MDSCs in blood after co-immunization with pCAG-GM. Unexpectedly, there was no significant difference in the number of MDSCs in pCAG-JME-immunized mice with or without pCAG-GM (data not shown), implying that MDSCs were not involved in the induction of immune suppression by the GM-CSF plasmid in this study, and GM-CSF treatment may directly affect the maturation state of DCs and induce the generation of Tregs, thereby resulting in immune suppression.

A study by Serafini [35] demonstrated that CD11b^+^Gr-1^+^ MDSCs were responsible for the induction of T-cell dysfunctions and were associated with the temporary impairment of T-lymphocyte reactivity. Additionally, Parmiani [31] analyzed studies dealing with the immune adjuvant activity of GM-CSF both in animal models and in clinical trials and concluded that GM-CSF may increase the vaccine-induced immune response when repeatedly administered at relatively low doses, whereas an opposite effect was often reported at high doses. According to these results, the lack of obvious changes in MDSC numbers in our study may be associated with the dose of GM-CSF used in our study, which may have failed to achieve a systemic concentration high enough to activate MDSCs.

In summary, we have shown that co-inoculation of the GM-CSF plasmid suppressed both cellular- and Ab-specific responses induced by JEV prM-E DNA vaccination. Additionally, Th1 immune responses were more sensitive to GM-CSF treatment than was Th2. The immune suppression was dose- and timing- dependent and related to the antigens. This effect was accompanied by an increase in IL-10, the mobilization of DCs with a tolerogenic phenotype and an expansion of Tregs but not MDSCs, suggesting that immature DCs, IL-10 and Tregs are involved in the immune suppression induced by co-inoculation of JEV prM-E DNA with pCAG-GM. The results provided useful information not only for further understanding of the versatility of GM-CSF function but also for development of safe clinical applications. Moreover, as already discussed, GM-CSF has opposite effects on the immune response induced by different DNA vaccines under different mechanisms, leading either to the amplification or down-regulation of the immune reactions. In the near future, it will be of paramount importance to establish under which conditions GM-CSF enhances the immune response or induces immune suppression to optimize its potential clinical use as a vaccine adjuvant or as a therapeutic agent.

## Materials and Methods

### Ethics Statement

This study was carried out in strict accordance with the recommendations in the national guidelines for the use of animals in scientific research “Regulations for the Administration of Affairs Concerning Experimental Animals”. The protocol was also approved by the Animal Care and Use Committee of Chinese Capital Medical University (Permit Number 2009-X-870). All surgeries were performed under diethyl ether anesthesia, and all efforts were made to minimize suffering.

### Mice, cells and viruses

Female inbred BALB/c mice were purchased from the Laboratory Animal Center of the Academy of Military Medical Sciences (Beijing, China) and maintained at specific-pathogen-free conditions.

*Aedes albopictus* cells (C6/36, ATCC CRL-1660) were grown at 28°C in RPMI 1640 (GIBCO, USA) supplemented with 10% fetal bovine serum (FBS). African green monkey kidney cells (Vero, ATCC CRL-1586) were grown at 37°C in Minimal Essential Medium (GIBCO, USA) supplemented with 5% FBS.

The JEV (Beijing-1 strain), the DENV1 (Hawaii strain) and the DENV2 (TR1751 strain) were propagated in C6/36 cell cultures and stored at −70°C until use. Virus titers were determined by the standard plaque assay on Vero cells under 1.3% methylcellulose overlay medium.

### Plasmids

The eukaryotic expression vector pCAGGSP7, containing a ubiquitously strong β-actin promoter and allowing efficient selection for transfectants that express foreign genes at high levels [42], was a parental plasmid used for the construction of DNA vaccines or the adjuvant plasmid and served as a control in this study.

The pCAG-JME DNA vaccine was constructed for the expression of JEV prM-E using the vector pCAGGSP7. Briefly, genomic RNA of JEV was extracted from JEV (Bejing-1)-infected C6/36 cells and served as a template for RT-PCR. The JEV prM-E fragment containing the prM signal sequence (spanning nucleotides 408 to 2477) obtained by RT-PCR was digested with the Xho I and Not I restriction enzymes (MBI Fermentas, USA), and subcloned into the multiple cloning site of pCAGGSP7. Similarly, plasmids expressing prM-E of DENV 1 (nucleotides 365 to 2419) and DENV 2 (nucleotides 367 to 2421) were constructed as described previously [43]. Plasmids expressing Core (nucleotides 332 to 904) and E1 (nucleotides 851 to 1480) fragments of HCV (HC-J4-91) were constructed following the same protocol.

The pCAG-GM plasmid encoding the murine GM-CSF fragment was constructed following a previously described protocol [10]. All gene sequences were obtained from GenBank Database (http://www.ncbi.nlm.nih.gov/genbank).

The recombinant plasmids were confirmed by DNA sequencing (TaKaRa, China) and double enzyme digestion, and the expression of the plasmids was further confirmed by indirect IFA in Vero cells. For immunization, the plasmid DNA was extracted and purified from the transformed *Escherichia coli* strain JM109 with an endotoxin-free plasmid extraction kit (Omega, USA). The purified plasmids were then reconstituted in sterile saline at a concentration of 1.0 mg/ml prior to use.

### Experimental design

For DNA immunization, six-week-old female BALB/c mice were pretreated with 50 µl 0.25% Lidocaine hydrochloride in each quadriceps muscle two days before the first DNA inoculation to enhance the muscle-cell uptake of plasmid DNA [44]. Groups of mice were then injected intramuscularly three times at three-week intervals with a mixture of 50 µg pCAG-GM and 50 µg pCAG-JME or 50 µg pCAG-JME and 50 µg pCAGGSP7 in 100 µl sterile saline. Mice immunized with 100 µg pCAGGSP7 vector alone served as negative controls. Three weeks after the final immunization, serum samples were collected for the evaluation of the pre-challenge serum Ab titers.

To determine if the expression level of GM-CSF was related with the inoculated amount, the mice were injected with various doses of pCAG-GM (10, 25 or 50 µg) or pCAGGSP7 (50 µg) and expressed levels of GM-CSF in the undiluted sera were measured by ELISA at pre-inoculation and 1, 3, 5, 7 and 9 days post-inoculation using mouse GM-CSF ELISA kit (R&D Systems, USA), according to the manufacture's direction. Five mice were used for each dose and each time point.

To investigate if the GM-CSF effect was dose-dependent, five groups of mice were immunized three times with the pCAG-JME plasmid (50 µg) plus various doses of pCAG-GM (10, 25 or 50 µg) or pCAGGSP7 (50 µg) at three-week intervals; anti-JEV-prM-E Ab levels were then measured.

Similarly, to determine if the GM-CSF effect was timing-dependent, mice were divided into six groups, and pCAG-GM was injected 3 days or 1 day prior to, simultaneously or 1 day after pCAG-JME immunization at the same location, and anti-JEV-prM-E Ab levels were analyzed after two boosters.

To analyze if the effects of GM-CSF were antigen-dependent, DNA immunizations were also performed using 50 µg pCAG-D1ME, pCAG-D2ME, pCAG-HCV-C or pCAG-HCV-E1 with 50 µg pCAG-GM or pCAGGSP7 following the procedures as was used for the pCAG-JME immunizations. After the final immunization, Ab titers were measured by ELISA.

For the protection test, three weeks after the third vaccination, the mice were challenged intraperitoneally with JEV (Beijing-1) at a lethal dose (50 LD50), followed by a sham intracerebral injection, which served to increase the susceptibility of animals to a central nervous system infection [45]. These mice were observed daily for symptoms of viral encephalitis and mortality over 21 days.

### Measurement of serum Ab levels by ELISA

Mice serum samples were collected by tail bleeding at different time points and were tested for anti- JEV-prM-E Ab responses by ELISA. Briefly, 96-well microtiter plates were coated overnight at 4°C with 10 µg/well of the concentrated virus proteins in 100 µl carbonate-bicarbonate buffer (pH 9.6). The plates were washed with phosphate-buffered saline (PBS) containing 0.2% Tween-20 (PBS-T) and blocked at 37°C for 1 h with PBS containing 1% bovine serum albumin (BSA). Serum samples (100 µl) were then added in two-fold serial dilutions (from 1∶100) and incubated at 37°C for 1 h. After washing with PBS-T, the plates were incubated at 37°C for 1 h with 100 µl goat anti-mouse IgG-HRP (1∶3000, KPL, USA) followed by the addition of the orthophenylene diamine substrate solution for visualization. The reactions were stopped by the addition of 2 M H_2_SO_4_. The OD values were measured at 492 nm with an ELISA microplate reader (Thermo Scientific, USA). The end-point titers were defined as the reciprocal of the highest serum dilution that yielded an OD value twice equal to or greater than the mean OD values of negative control samples. For the measurement of the IgG subclasses, goat anti-mouse IgG1-HRP and IgG2a-HRP (1∶1000, SBA, USA) were used as probes instead of IgG-HRP. Samples were tested in duplicate and repeated at least twice.

In addition, the levels of anti-DENV-1 or DENV-2 Abs were also detected with the same method, except DENV-1 or DENV-2 proteins replaced the JEV proteins as the antigens. The levels of anti-HCV IgG were determined by ELISA kit (CSB, China) following the manufacturer's instructions.

### Indirect Ab IFA

Anti-JEV Ab levels were also evaluated by indirect IFA three weeks after the third inoculation. Briefly, monolayers of Vero cells on sterile glass cover slips were infected with JEV for 21 h and used as the antigen. Cells were washed with PBS, fixed with 4% paraformaldehyde for 20 min, permeabilized with 0.2% Triton X-100 in PBS for 5 min and then blocked with 1% BSA in PBS for 1 h. After incubation with DNA-immunized or normal mouse serum (1∶300) at 4°C overnight, the cell cover slips were washed and incubated with FITC-conjugated goat anti-mouse IgG (1∶200, Immunotech, France) for 1 h. A cover slip incubated with mouse anti-JEV E glycoprotein mAb (1∶100, Abcam, USA) served as a positive control. After washing, air-drying and mounting, the cells were examined and photographed under a fluorescence microscope (Olympus BX61, Japan).

### Cytokine assay

Splenocytes isolated from mice three weeks after the final vaccination were subjected to the cytokine assay using IFN-γ, IL-2, IL-4 (BD Biosciences, USA), IL-10 and IL-17 ELISPOT sets (R&D Systems, USA), according to the manufacturer's instructions. In brief, splenocytes were aliquoted at 1×10^6^ per well into 96-well MultiScreen HTS Filter Plates (Millipore, USA) pre-coated with anti-mouse IFN-γ, IL-2, IL-4, IL-10 and IL-17 capture Abs, followed by stimulation with concentrated JEV proteins at 5 µg/well for 48 h at 37°C. Simultaneously, the splenocytes were co-cultured with concanavalin A as a positive control or with RPMI 1640 medium alone as a negative control. After incubation with biotin-conjugated secondary Abs and streptavidin-HRP (for the IFN-γ, IL-2 and IL-4 assay), single cytokine-positive cells were visualized by adding AEC substrate and counted using an ELISPOT reader (CTL, USA) with the Immunospot image analyzer software version 4.0. For the IL-10 and IL-17 assay, streptavidin-AP and BCIP/NBT chromogen were used instead of streptavidin-HRP and AEC substrate, respectively.

### FACS analysis of DC maturation in peripheral blood

DC maturation in peripheral blood was determined by a surface marker staining assay with direct FACS analysis on the 8th day after the final immunization. Rat anti-mouse CD16/CD32 mAb (clone 2.4G2), PE-conjugated hamster anti-mouse CD11c (clone HL3) mAb, APC-conjugated rat anti-mouse CD40 (clone 3/23), CD80 (clone 16-10A1) mAbs, hamster anti-mouse CD86 (clone GL1) mAb and subclass-matched control Abs were purchased from BD Pharmingen (USA). APC-conjugated rat mAb to mouse MHC II was purchased from Miltenyi Biotec (Germany).

100 µl blood taken by retro-orbital sinus puncture under anesthesia with diethyl ether was collected into a heparin-coated tube then blocked with rat anti-mouse CD16/CD32 mAb for 15 min at 4°C to eliminate non-specific binding to Fc receptors. Cells were stained sequentially with purified anti-mouse CD11c, CD40, CD80, CD86 and MHC II mAbs or subclass-matched control Abs for 30 min at room temperature followed by treatment with FACS Lysing Solution (BD Biosciences, USA) for 10 min, which lysed the erythrocytes and fixed other cells. Cells were washed twice and resuspended in 200 µl 1% paraformaldehyde. Samples were run on a FACSCalibur flow cytometer (BD Biosciences, USA) and analyzed using CellQuest Pro software (version 6.0).

### FACS analysis of CD4^+^CD25^+^Foxp3^+^ Tregs in peripheral blood

Three weeks after the final immunization, Tregs in peripheral blood were analyzed by FACS. PerCP-conjugated hamster anti-mouse CD3e (clone 145-2C11) mAb, FITC-conjugated rat anti-mouse CD4 (clone RM4-5) mAb and APC-conjugated rat anti-mouse CD25 (clone PC61) mAb were purchased from BD Pharmingen (USA). PE-conjugated rat anti-mouse Foxp3 mAb (clone FJK-16 s) was from eBioscience (USA).

100 µl heparinized blood were blocked with rat anti-mouse CD16/CD32 mAb for 15 min at 4°C and then incubated with anti-mouse CD3e, CD4, CD25 mAbs or their isotype controls for surface marker staining. Subsequently, the cells were treated with FACS lysing solution for 10 min. After washing, fixation, permeabilization and a second blocking, intracellular labeling of Foxp3 protein was performed by treating cells with anti-mouse Foxp3 mAb for 30 min on ice in the dark. Cells were washed twice and resuspended in 200 µl PBS containing 1% FBS followed by immediate flow cytometric analysis.

### FACS analysis of MDSCs in peripheral blood

100 µl heparinized blood was collected on day 5, 8 and 11 after the final immunization followed by blocking and direct staining of cell-surface markers. The following Abs purchased from BD Pharmingen were used: PE-conjugated rat anti-mouse CD11b (clone M1/70) mAbs, FITC-conjugated rat anti-mouse Ly6G and Ly6C (clone RB6-8C5) mAbs and isotype control Abs.

### Analysis of the expression level of JEV prM-E

The expression of JEV prM-E was detected by ELISA in mice serum samples at pre-inoculation and 1, 3, 5, 7, 14 and 21 days post-inoculation. The 96-well microtiter plates were coated overnight at 4°C with 10 µl of each serum sample diluted with 90 µl carbonate-bicarbonate buffer (pH 9.6). The mouse anti-JEV E glycoprotein mAb (Abcam, USA) was used as the primary Ab at a 1∶50 dilution. The other procedures were as the same as described above.

### Statistical analysis

Statistical analysis was performed using SPSS 17.0 software. Survival curves were compared with log-rank test. Others were compared using Student t test or one-way ANOVA test. Data were considered statistically significant if *p*<0.05.

## References

[1] Kaufman HL, Flanagan K, Lee CSD, Perretta DJ, Horig H (2002). Insertion of interleukin-2 (IL-2) and interleukin-12 (IL-12) genes into vaccinia virus results in effective anti-tumor responses without toxicity.. Vaccine.

[2] Chakrabarti R, Chang Y, Song K, Prud'homme GJ (2004). Plasmids encoding membrane-bound IL-4 or IL-12 strongly costimulate DNA vaccination against carcinoembryonic antigen (CEA).. Vaccine.

[3] Abaitua F, Rodríguez JR, Garzóna A, Rodrígueza D, Esteban M (2006). Improving recombinant MVA immune responses: potentiation of the immune responses to HIV-1 with MVA and DNA vectors expressing Env and the cytokines IL-12 and IFN-gamma.. Virus Res.

[4] Nimal S, Heath AW, Thomas MS (2006). Enhancement of immune responses to an HIV gp120 DNA vaccine by fusion to TNF alpha cDNA.. Vaccine.

[5] Zhang X, Divangahi M, Ngai P, Santosuosso M, Millar J (2007). Intramuscular immunization with a monogenic plasmid DNA tuberculosis vaccine: Enhanced immunogenicity by electroporation and co-expression of GM-CSF transgene.. Vaccine.

[6] Chakrabarti R, Zhou ZF, Chang Y, Prud'homme GJ (2005). A mutant B7-1/Ig fusion protein that selectively binds to CTLA-4 ameliorates anti-tumor DNA vaccination and counters regulatory T cell activity.. Vaccine.

[7] Chang SY, Lee K-C, Ko S-Y, Ko H-J, Kang C-Y (2004). Enhanced efficacy of DNA vaccination against Her-2/neu tumor antigen by genetic adjuvants.. Int J cancer.

[8] Xu R, Megati S, Roopchand V, Luckay A, Masood A (2008). Comparative ability of various plasmid-based cytokines and chemokines to adjuvant the activity of HIV plasmid DNA vaccines.. Vaccine.

[9] Loudon PT, Yager EJ, Lynch DT, Narendran A, Stagnar C (2010). GM-CSF increases mucosal and systemic immunogenicity of an H1N1 influenza DNA vaccine administered into the epidermis of non-human primates.. PLoS ONE.

[10] Gao N, Chen W, Zheng Q, Fan D-y, Zhang J-l (2010). Co-expression of Japanese encephalitis virus prM-E-NS1 antigen with granulocyte-macrophage colony-stimulating factor enhances humoral and anti-virus immunity after DNA vaccination.. Immunol Lett.

[11] Qin L, Greenland JR, Moriya C, Cayabyab MJ, Letvin NL (2007). Effects of type I interferons on the adjuvant properties of plasmid granulocyte-macrophage colony-stimulating factor in vivo.. J Virol.

[12] Slingluff CL, Petroni GR, Olson WC, Smolkin ME, Ross MI (2009). Effect of granulocyte/macrophage colony-stimulating factor on circulating CD8^+^ and CD4^+^ T-cell responses to a multipeptide melanoma vaccine: outcome of a multicenter randomized trial.. Clin Cancer Res.

[13] Ramanathan RK, Potter DM, Belani CP, Jacobs SA, Gravenstein S (2002). Randomized trial of influenza vaccine with granulocyte-macrophage colony-stimulating factor or placebo in cancer patients.. J Clin Oncol.

[14] Shi Y, Liu CH, Roberts AI, Das J, Xu G (2006). Granulocyte-macrophage colony-stimulating factor (GM-CSF) and T-cell responses: what we do and don't know.. Cell Res.

[15] Lahoud MH, Proietto AI, Gartlan KH, Kitsoulis S, Curtis J (2006). Signal regulatory protein molecules are differentially expressed by CD8- dendritic cells.. J Immunol.

[16] Banchereau J, Steinman RM (1998). Dendritic cells and the control of immunity.. Nat Immunol.

[17] Mahnke K, Schmitt E, Bonifaz L, Enk AH, Jonuleit H (2002). Immature, but not inactive: the tolerogenic function of immature dendritic cells.. Immunol Cell Biol.

[18] Lutz MB, Schuler G (2002). Immature, semi-mature and fully mature dendritic cells: which signals induce tolerance or immunity?. Trends Immunol.

[19] Maldonado RA, von Andrian UH (2010). How tolerogenic dendritic cells induce regulatory T cells.. Adv Immunol.

[20] Mahnke K, Bedke T, Enk AH (2007). Regulatory conversation between antigen presenting cells and regulatory T cells enhance immune suppression.. Cell Immunol.

[21] Ganesh BB, Cheatem DM, Sheng JR, Vasu C, Prabhakar BS (2009). GM-CSF-induced CD11c+CD8a-dendritic cells facilitate Foxp3^+^ and IL-10^+^ regulatory T cell expansion resulting in suppression of autoimmune thyroiditis.. Int Immunol.

[22] Sheng JR, Li L, Ganesh BB, Vasu C, Prabhakar BS (2006). Suppression of experimental autoimmune myasthenia gravis by granulocyte-macrophage colony-stimulating factor is associated with an expansion of FoxP3^+^ regulatory Tcells.. J Immunol.

[23] Gaudreau S, Guindi C, Me'nard Ml, Besin G, Dupuis G (2007). Granulocyte-macrophage colony-stimulating factor prevents diabetes development in NOD mice by inducing tolerogenic dendritic cells that sustain the suppressive function of CD4^+^CD25^+^ regulatory T cells.. J Immunol.

[24] Serafini P, De Santo C, Marigo I, Cingarlini S, Dolcetti L (2004). Derangement of immune responses by myeloid suppressor cells.. Cancer Immunol Immunother.

[25] Morales JK, Kmieciak M, Knutson KL, Bear HD, Manjili MH (2010). GM-CSF is one of the main breast tumor-derived soluble factors involved in the differentiation of CD11b^−^Gr1^−^ bone marrow progenitor cells into myeloid-derived suppressor cells.. Breast Cancer Res Treat.

[26] Kared H, Leforban B, Montandon R, Renand A, Layseca Espinosa E (2008). Role of GM-CSF in tolerance induction by mobilized hematopoietic progenitors.. Blood.

[27] Hirota K, Martin B, Veldhoen M (2010). Development, regulation and functional capacities of Th17 cells.. Semin Immunol.

[28] Gangi E, Vasu C, Cheatem D, Prabhakar BS (2005). IL-10-producing CD4^+^CD25^+^ regulatory T cells play a critical role in granulocyte-macrophage colony-stimulating factor-induced suppression of experimental autoimmune thyroiditis.. J Immunol.

[29] Jonuleit H, Schmitt E, Schuler G, Knop J, Enk AH (2000). Induction of interleukin 10-producing, nonproliferating CD4(+) T cells with regulatory properties by repetitive stimulation with allogeneic immature human dendritic cells.. J Exp Med.

[30] Chen HW, Pan CH, Huan HW, Liau MY, Chiang JR (2001). Suppression of immune response and protective immunity to a Japanese encephalitis virus DNA vaccine by coadministration of an IL-12-expressing plasmid.. J Immunol.

[31] Parmiani G, Castelli C, Pilla L, Santinami M, Colombo MP (2007). Opposite immune functions of GM-CSF administered as vaccine adjuvant in cancer patients.. Ann Oncol.

[32] Mach N, Gillessen S, Wilson SB, Sheehan C, Mihm M (2000). Differences in dendritic cells stimulated in vivo by tumors engineered to secrete granulocyte-macrophage colony-stimulating factor or Flt3-ligand.. Cancer Res.

[33] Nemunaitis J (2005). Vaccines in cancer: GVAX, a GM-CSF gene vaccine.. Expert Rev Vaccines.

[34] Gillessen S, Naumov YN, Nieuwenhuis EE, Exley MA, Lee FS (2003). CD1d-restricted T cells regulate dendritic cell function and antitumor immunity in a granulocyte-macrophage colony-stimulating factor-dependent fashion.. Proc Natl Acad Sci U S A.

[35] Serafini P, Carbley R, Noonan KA, Tan G, Bronte V (2004). High-dose granulocyte-macrophage colony-stimulating factor-producing vaccines impair the immune response through the recruitment of myeloid suppressor cells.. Cancer Res.

[36] Kusakabe K, Xin KQ, Katoh H, Sumino K, Hagiwara E (2000). The timing of GM-CSF expression plasmid administration influences the Th1/Th2 response induced by an HIV-1-specific DNA vaccine.. J Immunol.

[37] Rutella S, Lemoli RM (2004). Regulatory T cells and tolerogenic dendritic cells: from basic biology to clinical applications.. Immunol Lett.

[38] Grcevic D, Lukic IK, Kovacic N, Ivcevic S, Katavic V (2006). Activated T lymphocytes suppress osteoclastogenesis by diverting early monocyte/macrophage progenitor lineage commitment towards dendritic cell differentiation through down-regulation of receptor activator of nuclear factor-kappaB and c-Fos.. Clin Exp Immunol.

[39] Wallet MA, Sen P, Tisch R (2005). Immunoregulation of dendritic cells.. Clin Med Res.

[40] Song X, Liang F, Liu N, Luo Y, Xue H (2009). Construction and characterization of a novel DNA vaccine that is potent antigen-specific tolerizing therapy for experimental arthritis by increasing CD4^+^CD25^+^Treg cells and inducing Th1 to Th2 shift in both cells and cytokines.. Vaccine.

[41] Dolcetti L, Peranzoni E, Ugel S, Marigo I, Fernandez Gomez A (2010). Hierarchy of immunosuppressive strength among myeloid-derived suppressor cell subsets is determined by GM-CSF.. Eur J Immunol.

[42] Niwa H, Yamamura K, Miyazaki J (1991). Efficient selection for high-expression transfectants with a novel eukaryotic vector.. Gene.

[43] Zheng Q, Fan D, Gao N, Chen H, Wang J (2011). Evaluation of a DNA vaccine candidate expressing prM-E-NS1 antigens of dengue virus serotype 1 with or without granulocyte-macrophage colony-stimulating factor (GM-CSF) in immunogenicity and protection.. Vaccine.

[44] Yang B, Lan X, Li X, Yina X, Baoyu Lia XH (2008). A novel bi-functional DNA vaccine expressing VP1 protein and producing antisense RNA targeted to 5′UTR of foot-and-mouth disease virus can induce both rapid inhibitory effect and specific immune response in mice.. Vaccine.

[45] Chen H-W, Pan C-H, Liau M-Y, Jou R, Tsai C-J (1999). Screening of protective antigens of Japanese encephalitis virus by DNA immunization: a comparative study with conventional viral vaccines.. J Virol.

